# The α-d-anomer of 2′-de­oxy­cyti­dine: crystal structure, nucleo­side conformation and Hirshfeld surface analysis

**DOI:** 10.1107/S2053229621003430

**Published:** 2021-04-09

**Authors:** Simone Budow-Busse, Yingying Chai, Sebastian Lars Müller, Constantin Daniliuc, Frank Seela

**Affiliations:** a Laboratory of Bioorganic Chemistry and Chemical Biology, Center for Nanotechnology, Heisenbergstrasse 11, 48149 Münster, Germany; b Organisch-Chemisches Institut, Westfälische Wilhelms-Universität Münster, Corrensstrasse 40, 48149 Münster, Germany; c Laboratorium für Organische und Bioorganische Chemie, Institut für Chemie neuer Materialien, Universität Osnabrück, Barbarastrasse 7, Osnabrück 49069, Germany

**Keywords:** α-2′-de­oxy­cyti­dine, crystal structure, crystal packing, Hirshfeld surface analysis, anomer, nucleic acid chemistry

## Abstract

β-2′-De­oxy­ribo­nucleo­sides are the constituents of nucleic acids, whereas their anomeric α-analogues are rarely found in nature. Moreover, not much infor­mation is available on the structural and conformational parameters of α-2′-de­oxy­ribo­nucleo­sides. This study reports on the single-crystal X-ray structure of α-2′-de­oxy­cyti­dine, C_9_H_13_N_3_O_4_ (**1**), and the conformational param­eters characterizing **1** were determined. The conformation at the glycosylic bond is *anti*, with χ = 173.4 (2)°, and the sugar residue adopts an almost symmetrical C2′-*endo*-C3′-*exo* twist (; *S*-type), with *P* = 179.7°. Both values lie outside the conformational range usually preferred by α-nucleo­sides. In addition, the amino group at the nucleobase shows partial double-bond character. This is supported by two separated signals for the amino protons in the ^1^H NMR spectrum, indicating a hindered rotation around the C4—N4 bond and a relatively short C—N bond in the solid state. Crystal packing is controlled by N—H⋯O and O—H⋯O contacts between the nucleobase and sugar moieties. Moreover, two weak C—H⋯N contacts (C1′—H1′ and C3′—H3′*A*) are observed. A Hirshfeld surface analysis was carried out and the results support the inter­molecular inter­actions observed by the X-ray analysis.

## Abstract 2

α-2′-De­oxy­cyti­dine is the α-anomeric analogue of the canonical β-2′-de­oxy­cyti­dine and adopts conformational parameters which lie outside the conformational range usually preferred by α-nucleo­sides. Crystal packing is controlled by N—H⋯O and O—H⋯O contacts between the nucleobase and sugar moieties.

## Introduction   

Nucleosides with an α-configuration at the anomeric carbon are seldom found in nature (Ni *et al.*, 2019 ▸). α-Nucleosides are not building blocks of naturally occurring DNA or RNA. However, α-nucleo­sides have been isolated as constituents of small mol­ecules in living cells, such as vitamin B_12_ (Bonnett, 1963 ▸) or a nicotinamide adenine dinucleotide (NAD) derivative isolated from *Azobacter vinelandii* (Suzuki *et al.*, 1965 ▸). Also, chemical nucleo­side synthesis yields α-nucleo­sides together with the β-anomers in ratios depending on the structures of the starting materials and the experimental conditions. Protocols were developed for the stereoselective synthesis of α-d nucleo­sides or by anomerization of β-d anomers. This topic has been reviewed recently by Ni *et al.* (2019 ▸).

α-Nucleosides were also incorporated into oligonucleotides, replacing single β-nucleo­sides (Guo & Seela, 2017 ▸), or α-oligo­nucleotides were constructed which are entirely com­posed of α-nucleo­sides (Morvan *et al.*, 1990 ▸). α-Oligonucleotides form duplexes with an anti­parallel orientation, with com­plementary strands also having an α-configuration (Morvan *et al.*, 1987*a* ▸), while duplexes with a parallel alignment are formed when the com­plementary strand is a β-oligonucleotide (Morvan *et al.*, 1987*b* ▸).

Recently, we reported on the stability and recognition of silver-mediated heterochiral DNA with com­plementary α/β-strands (Chai *et al.*, 2020 ▸). Also, silver-mediated homochiral duplexes were constructed in which single residues were replaced by α-dC (**1**) (Scheme 1) (Guo & Seela, 2017 ▸). The silver-mediated base pair formed by anomeric α-dC (**1**) with β-dC (**2**) shows significantly higher stability than that formed by the silver-mediated β-dC–β-dC pair. Not only the stability of the metal-mediated base pair is higher, but also the metal-free α-dC–β-dC mismatch is more stable.

Conformational studies on α-nucleo­sides revealed distinct differences com­pared to their β-anomeric counterparts (Sundaralingam, 1971 ▸; Latha & Yathindra, 1992 ▸). In general, the flexibility around the glycosylic linkage, as well as the sugar pucker of α-nucleo­sides, seems to be more restricted than for β-nucleo­sides. The different conformational properties of the α/β-anomers were attributed to the differences in the steric inter­actions between the nucleobase and the sugar moiety (Sundaralingam, 1971 ▸; Latha & Yathindra, 1992 ▸). However, com­pared to the number of X-ray analyses of β-nu­cleo­sides, studies on α-nucleo­sides are extremely limited (Sundaralingam, 1971 ▸; Latha & Yathindra, 1992 ▸). Surprisingly, among the canonical α-nucleo­sides, only the solid-state conformations of α-cyti­dine (Post *et al.*, 1977 ▸) and α-2′-de­oxy­thy­midine (**3**) (Görbitz *et al.*, 2005 ▸) have been reported. Moreover, some X-ray studies on modified α-nucleo­side analogues have been reported, *e.g.* α-5-acetyl-2′-de­oxy­uridine (Hamor *et al.*, 1977 ▸), α-5-aza-7-de­aza-2′-de­oxy­guanosine (Seela *et al.*, 2002 ▸), α-5-iodo-2′-de­oxy­cyti­dine (Müller *et al.*, 2019 ▸) and α-5-octa­diynyl-2′-de­oxy­cyti­dine (Zhou *et al.*, 2019 ▸). Among the α-2′-de­oxy­ribo­nucleo­sides, the solid-state conformations of the α-anomers of 2′-de­oxy­cyti­dine (**1**), 2′-de­oxy­adenosine and 2′-de­oxy­guanosine are still unknown.
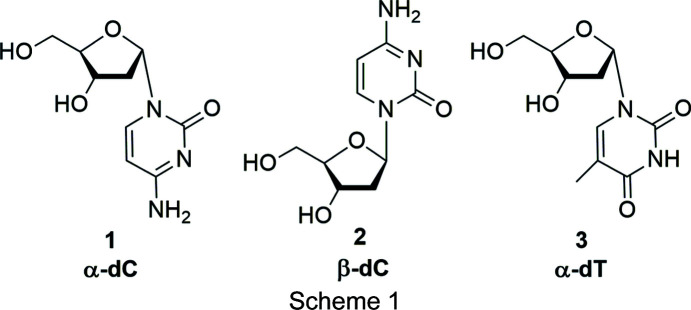


The single-crystal X-ray analysis of α-2′-de­oxy­cyti­dine (**1**) was performed in order to obtain a deeper insight into the conformational properties of **1** in the solid-state. This is the second report of an α-anomer of a canonical pyrimidine 2′-de­oxy­ribo­nucleo­side besides α-2′-de­oxy­thymidine (**3**) (Gör­bitz *et al.*, 2005 ▸). The results are com­pared to the structure of β-dC (Young & Wilson, 1975 ▸). For both **1** and **2**, the sugar conformation in solution was determined using a ^1^H NMR-based method. Moreover, a Hirshfeld surface analysis of **1** was carried out to visualize the packing inter­actions.

## Experimental   

### Synthesis and crystallization of α-dC (1)   

α-2′-De­oxy­cyti­dine (**1**) was synthesized as reported pre­viously (Chai *et al.*, 2019 ▸). For crystallization, com­pound **1** was dissolved in methanol containing 10% water (10 mg in 1 ml) and was obtained as colourless prisms (m.p. 203–204 °C; Yamaguchi & Saneyoshi, 1984 ▸) by slow evaporation of the solvent at room temperature. A colourless prism-like speci­men of **1** was used for the X-ray crystallographic analysis.

### Refinement   

Crystal data, data collection and structure refinement details are summarized in Table 1 ▸. The H atoms on N4, O3′ and O5′ were refined freely.

## Results and discussion   

### Mol­ecular geometry and conformation of α-dC (1)   

**Figure 1:**
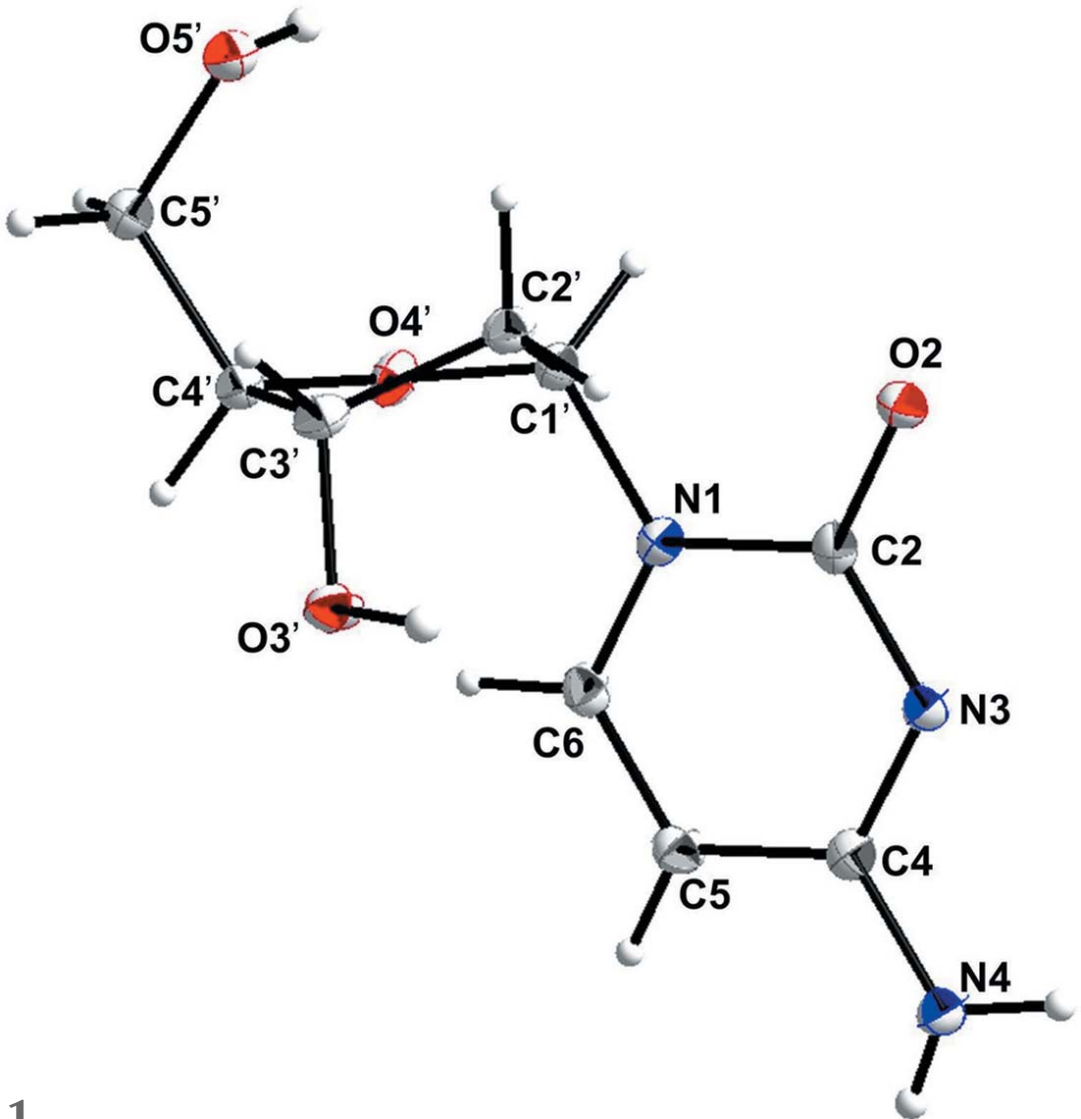
Perspective view of the α-d anomer of 2′-de­oxy­cyti­dine (**1**), showing the atomic numbering scheme. Displacement ellipsoids are drawn at the 50% probability level and H atoms are shown as small spheres of arbitrary size.

The three-dimensional (3D) structure of α-dC (**1**) is shown in Fig. 1 ▸ and selected geometric parameters are presented in Table 2 ▸. The 3D structure of **1** clearly indicates the α-orientation of the nucleobase (Fig. 1 ▸), which in addition is supported by the Flack parameter (see Table 1 ▸; Parsons *et al.*, 2013 ▸). Moreover, according to the synthetic pathway, the anomeric centre at C1′ shows an *S*-configuration, confirming the α-d anomeric structure of **1**.

The crystal structure of the related canonical β-2′-de­oxy­cyti­dine (**2**) has been reported previously (Young & Wilson, 1975 ▸). The β-anomer **2** shows two conformers (**2a** and **2b**) in the unit cell. As not many single-crystal X-ray analyses of α-2′-de­oxy­ribo­nucleo­sides exist, it was of inter­est to com­pare the geometric parameters of the α/β-anomers of 2′-de­oxy­cyti­dine (**1** and **2**).

For pyrimidine nucleo­sides, the orientation of the nucleobase with respect to the sugar moiety (*syn*/*anti*) is defined by the torsion angle χ (O4′—C1′—N1—C2) (IUPAC–IUB Joint Commission on Biochemical Nomenclature, 1983 ▸). In the *anti* conformation, atom O2 of the six-membered ring is pointing away from the sugar, while in the *syn* conformation, O2 is pointing towards the sugar ring (Saenger, 1984 ▸). The preferred conformation for canonical pyrimidine β-2′-de­oxy­ribo­nucleo­sides, including β-dC (**2a** and **2b**), is *anti* (**2a**: χ = 201.2°; **2b**: χ = 222.2°) (Young & Wilson, 1975 ▸). In contrast to the broad range of *anti* conformations adopted by β-nucleo­sides, a rather narrow preferred *anti* range, together with a preference of χ to adopt lower *anti* values, has been reported for α-nucleo­sides (Latha & Yathindra, 1992 ▸). For instance, α-2′-de­oxy­thymidine (**3**) adopts a χ value of 124° (Görbitz *et al.*, 2005 ▸). However, in case of the title com­pound α-2′-de­oxy­cyti­dine (**1**), an *anti* conformation with χ = 173.39 (16)° is observed which is significantly greater. In addition, other solid-state structures of modified pyrimidine α-2′-de­oxy­ribo­nucleo­sides with χ values around 168° have been reported recently, which also fall into this range (Zhou *et al.*, 2019 ▸; Müller *et al.*, 2019 ▸).

The second conformational parameter of inter­est for nucleo­sides is the sugar puckering mode. The β-2′-de­oxy­ribo­furanosyl moiety shows a preference for two principal sugar conformations, namely C3′-*endo* (*N*) and C2′-*endo* (*S*) (Altona & Sundaralingam, 1972 ▸; Sundaralingam, 1971 ▸). In contrast, studies on α-2′-de­oxy- and ribo­nucleo­sides showed that these anomers prefer mainly C3′-*exo*, C2′-*exo* and C4′-*endo* conformations (Latha & Yathindra, 1992 ▸; Sundaralingam, 1971 ▸). As can be seen in Fig. 1 ▸, the sugar moiety of α-dC (**1**) adopts an almost symmetrical C2′-*endo*-C3′-*exo* twist (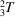; *S*-type), with a pseudorotational phase angle *P* = 179.7° and a maximum amplitude τ_m_ = 33.0° (Altona & Sundaralingam, 1972 ▸; Saenger, 1984 ▸). Thus, the α-2′-de­oxy­ribo­furanosyl moiety of **1** exhibits a C2′-*endo* conformation which is outside the preferred conformational range of α-2′-de­oxy­ribo­nucleo­sides. Other examples of α-2′-de­oxy­ribo­nucleo­sides with a C2′-*endo* conformation of the sugar residue include α-5-acetyl-2′-de­oxy­uridine (Hamor *et al.*, 1977 ▸) and the α-anomer of 5-aza-7-de­aza-2′-de­oxy­guanosine (Seela *et al.*, 2002 ▸). For com­parison, the conformers of the canonical β-dC (**2**) exhibit two different conformations, namely C3′-*endo* (*N*-type) for conformer **2a** and C2′-*endo* (*S*-type) for conformer **2b** (Young & Wilson, 1975 ▸).

The torsion angle γ (O5′—C5′—C4′—C3′) characterizes the orientation of the exocyclic 5′-hy­droxy group relative to the sugar ring (Saenger, 1984 ▸). Earlier studies on α-nucleo­sides indicate that the conformational preference about the C4′—C5′ bond is similar to that of β-nucleo­sides (Sundaralingam, 1971 ▸). For α-dC (**1**), γ is 55.9 (2)°, referring to a +*sc* (*gauche*, *gauche*) conformation which is similar to that found for β-dC (**2**) (56.7 and 62.5°; +*sc*, *gauche*, *gauche*) (Young & Wilson, 1975 ▸).

### Hydrogen bonding and mol­ecular packing of α-dC (1)   

**Figure 2:**
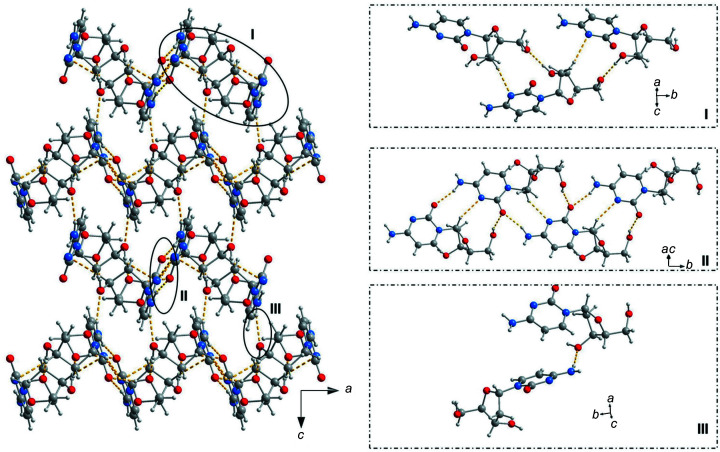
Crystal packing of α-2′-de­oxy­cyti­dine (**1**), shown along the *ac* plane (ball-and-stick model), and with magnifications of designated areas of the crystal packing, showing the hydrogen-bonding pattern.

**Figure 3:**
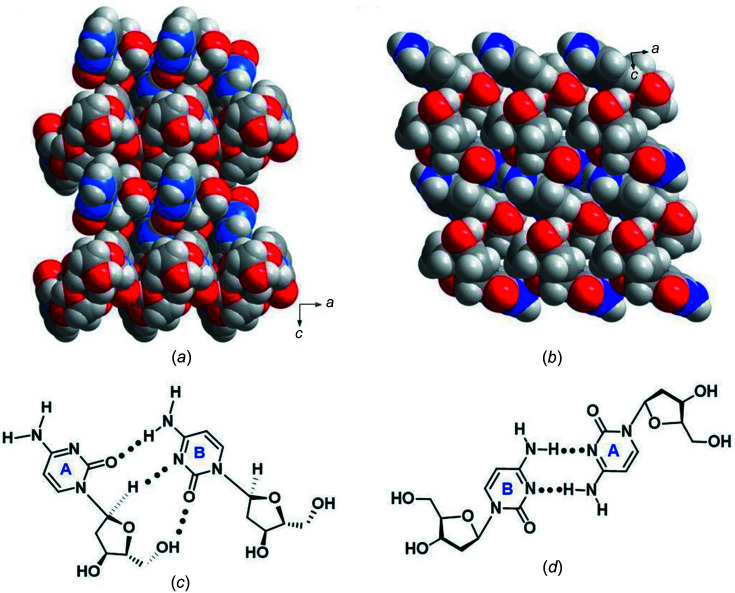
Space-filling models of (*a*) α-dC (**1**) and (*b*) β-dC (**2**). Schematic view of the inter­molecular hydrogen-bonding inter­actions of two nucleo­sides for (*c*) α-dC (**1**) and (*d*) β-dC (**2**).

Fig. 2 ▸ displays the crystal packing mode and hydrogen-bonding pattern for the crystal of α-dC (**1**). The corresponding hydrogen-bonding data and symmetry codes are summarized in Table 3 ▸. The particular nucleo­side units of **1** are connected by hydrogen bonds between (i) the nucleobases, (ii) nucleobases and sugars, as well as (iii) two sugar moieties. The crystal structure is formed by a repetition of nucleo­side units which are arranged in chains in a zigzag-like manner within the *ac* plane (Fig. 2 ▸). This arrangement is different to that of the canonical β-dC (**2**). Space-filling models of α-dC (**1**) and β-dC (**2**) shown in Figs. 3 ▸(*a*) and 3(*b*), respectively, visualize the different crystal packing modes.

In more detail, two cytosine residues of β-dC (**2**) (Young & Wilson, 1975 ▸) form a mismatch connected by two hydrogen bonds, each between atom N3 and the 4-amino group of the respective second mol­ecule (Fig. 3 ▸ *d*). Fig. 3 ▸(*c*) shows that the situation is com­pletely different for the α-anomer of 2′-de­oxy­cyti­dine (**1**). Only one hydrogen bond is formed between the nucleobases (N4—H4*B*⋯O2^ii^) (Fig. 2 ▸, motif II, and Table 3 ▸). The chains are further stabilized by a nucleobase-to-sugar contact (O5′—H5⋯O2^iii^; Fig. 2 ▸, motif II, and Table 3 ▸) and a sugar-to-sugar contact (O3′—H3′⋯O5′^v^; Fig. 2 ▸, motif I, and Table 3 ▸). In addition, two weak C—H⋯N contacts with C1′—H1′ (motif II) and C3′—H3′*A* (motif I) as the hydrogen-bond donors and N3 as the acceptor (N3^iii^ and N3^iv^, respectively) are observed. In most crystal structures of 2′-de­oxy­ribo­nucleo­sides, the C—H groups of the sugar moiety do not participate as hydrogen-bond donors in hydrogen bonding. However, a few examples have been reported, *e.g.* α-5-iodo-2′-de­oxy­cyti­dine (Müller *et al.*, 2019 ▸) and 7-iodo-5-aza-7-de­aza­guanosine (Kondhare *et al.*, 2020 ▸). Moreover, two neighbouring chains are connected by a N4—H4*A*⋯O3′ hydrogen bond, as illustrated in Fig. 2 ▸ (motif III), thereby generating a network.

### Hirshfeld surface analysis of α-dC (1)   

**Figure 4:**
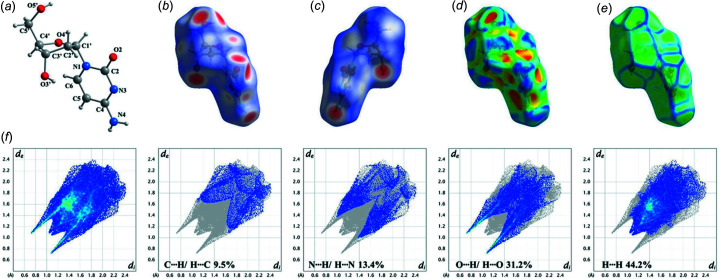
(*a*) Perspective view of α-dC (**1**), showing the atomic numbering scheme. The Hirshfeld surface of **1** mapped with (*b*) *d*_norm_ (−0.5 to 1.5, front view), (*c*) *d*_norm_ (−0.5 to 1.5, back view), (*d*) shape index and (*e*) curvedness, and (*f*) the corresponding fingerprint plots. Full inter­actions (left) and the resolved contacts (left, C⋯H/H⋯C; middle left, N⋯H/H⋯N; middle right, O⋯H/H⋯O; right, H⋯H) are shown, together with the percentages of their contribution to the total Hirshfeld surface area of α-anomer **1**.

To visualize the inter­molecular inter­actions of α-dC (**1**) in the solid-state, a Hirshfeld surface analysis was conducted and two-dimensional (2D) fingerprint plots were analysed (Spackman & Jayatilaka, 2009 ▸). The *CrystalExplorer* program (Version 17; Spackman & Jayatilaka, 2009 ▸; Turner *et al.*, 2017 ▸) was used to carry out the Hirshfeld surface analysis mapped over a *d*_norm_ range from −0.5 to 1.5 Å, shape index (−1.0 to 1.0 Å) and curvedness (−4.0 to 0.4 Å), as well as their associated 2D fingerprint plots (Fig. 4 ▸). On the *d*_norm_ surface of α-dC (**1**), several red areas (intense red spots) are observed (Figs. 4 ▸ *b* and 4 ▸ *c*), corresponding to the close contacts of the nucleobase and sugar residue (N—H⋯O and O—H⋯O). These inter­actions are shorter than the sum of the van der Waals radii and show negative *d*_norm_. Small and light-red coloured spots are also found (Fig. 4 ▸ *b*) and can be assigned to the weak contacts with C1′—H1′ and C3′—H3′*A* as hydrogen-bond donors and N3 as acceptor. The results of the Hirshfeld analyses are consistent with the hydrogen-bonding data (Table 3 ▸). The shape index (Fig. 4 ▸ *d*) indicates π–π stacking inter­actions by the presence of red and blue triangles, and flat surface patches within the curvedness surfaces (Fig. 4 ▸ *e*) are characteristic for planar stacking. However, as also indicated by Fig. 2 ▸, these inter­actions are less pronounced in the crystal structure of α-dC (**1**).

The 2D fingerprint plots provide a visual summary of the inter­molecular contacts in the crystal structure of **1** and can be resolved to particular atom-pair inter­actions and their relative contributions to the Hirshfeld surface, as illustrated in Fig. 4 ▸(*f*). Strong inter­actions are found for O⋯H/H⋯O (31.2%) and N⋯H/H⋯N (13.4%), which agrees with the fact that the crystal packing of α-dC (**1**) is largely controlled by N—H⋯O and O—H⋯O hydrogen bonds (Table 3 ▸).

### Conformation of the α- and β-anomers of 2′-de­oxy­cyti­dine in solution   

For canonical nucleo­sides with a β-d configuration, numer­ous studies exist describing their crystal structures and conformation in the solid-state and in solution. Compared to the information available for β-anomeric nucleo­sides, reports on α-anomers are limited (Poznański *et al.*, 2001 ▸). Moreover, the conformational change of anomeric nucleo­sides from β to α has an effect on the stability of the DNA double helix (Thibaudeau & Chattopadhyaya, 1997 ▸).

To ascertain the sugar conformation of α-dC (**1**) in solution, a conformational analysis of the furan­ose puckering of α-dC (**1**) and, for com­parison, of β-dC (**2**) was performed. To this end, high resolution (600 MHz) ^1^H NMR spectra were measured in dimethyl sulfoxide (DMSO) and coupling constants were determined (Table 4 ▸). The conformational analysis of the puckering of the 2′-de­oxy­ribo­furanosyl moiety was performed using the *PSEUROT* program (Version 6.3; Van Wijk *et al.*, 1999 ▸). This program calculates the population of *N*- and *S*-type conformers on the basis of five ^3^ *J*(H,H) coupling constants, namely, ^3^ *J*(H1′,H2′), ^3^ *J*(H1′,H2′′), ^3^ *J*(H2′,H3′), ^3^ *J*(H2′′,H3′) and ^3^ *J*(H3′,H4′). The coupling con­stants are summarized in Table 4 ▸ and the spectra are available in the supporting information.

**Figure 5:**
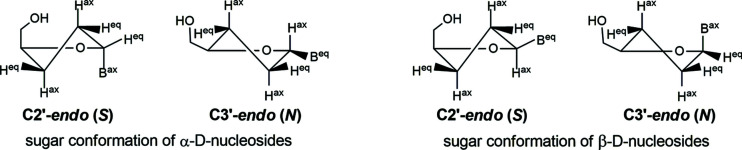
*N* and *S* conformations of α-d and β-d nucleo­sides in solution. ‘B’ corresponds to a nucleobase, with ax indicating axial and eq equatorial.

The *PSEUROT* analysis of α-dC (**1**) and β-dC (**2**) revealed that both nucleo­sides prefer an *S*-type sugar conformation (72 and 79% *S*-type, respectively) in solution. Accordingly, α-dC (**1**) adopts the same sugar conformation (*S*-type) in solution and the solid-state. The *S*-conformation is also the preferred conformation of the canonical sugar residues as constituents of DNA. In this regard, the sugar residue of α-nucleo­side **1** fits into the DNA backbone (Fig. 5 ▸).

Moreover, the ^1^H NMR spectra of α-dC (**1**) and β-dC (**2**) show, in both cases, two signals for the amino protons (Table 4 ▸ and Figs. S1 and S2 in the supporting information). The appearance of two separated resonances for the amino protons indicates a hindered rotation about the C4—N4 bond due to partial double-bond character. Moreover, in the solid-state structure of α-dC (**1**), the C4—N4 bond is relatively short [1.337 (3) Å; Table 2 ▸]. This suggests that the lone electron pair of the amino group is at least partially delocalized into the pyrimidine ring. These observations are consistent with earlier reports of 1-methyl­cytosine also reporting on the partial double-bond character of the amino group (Rossi & Kistenmacher, 1977 ▸; Fonseca Guerra *et al.*, 2014 ▸).

## Conclusion   

In this work, the crystal structure of the α-anomeric analogue of 2′-de­oxy­cyti­dine (**1**) has been studied. α-2′-De­oxy­ribo­nucleo­sides are not widespread in nature as they are not part of canonical DNA. Literature reports on the conformational properties of α-2′-de­oxy­ribo­nucleo­sides are also limited. The single-crystal X-ray analysis of α-2′-de­oxy­cyti­dine revealed conformational properties which are outside the preferred range of α-nucleo­sides. This is rather unexpected as α-dC (**1**) is a rather ‘simple’ α-nucleo­side without any further modifications at the nucleobase or sugar moiety. The *anti* conformation [χ = 173.39 (16)°] at the glycosylic bond is shifted to a higher χ value and the sugar moiety shows an almost symmetrical C2′-*endo*-C3′-*exo* twist (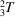; *S*-type), with *P* = 179.7°. The 2′-*endo* conformation is energetically less favoured in α-nucleo­sides com­pared to β-nucleo­sides, where this conformation is the preferred conformation of the DNA constituents. In addition, the C4—N4 bond between the amino group and the nucleobase is relatively short. Together with the appearance of two separated signals for the amino protons in the ^1^H NMR spectrum, this indicates a hindered rotation around the C4—N4 bond due to partial double-bond character. Within the crystal, the individual nucleo­side units of **1** are arranged in chains in a zigzag-like manner (*ac* plane). The crystal packing is controlled by N—H⋯O and O—H⋯O contacts between the nucleobase and sugar moieties. Moreover, two weak C—H⋯N contacts (C1′—H1⋯N3^iii^ and C3′—H3′*A*⋯N3^iv^) are observed.

Although the flexibility at the glycosylic bond and the sugar conformation are generally more restricted for α-nucleo­sides, α-2′-de­oxy­cyti­dine (**1**) is an example of an α-nucleo­side with properties found outside the energetically favoured conformational range. This work constitutes a useful contribution to the field of nucleic acid chemistry and expands the state of knowledge on α-nucleo­sides.

## Supplementary Material

Crystal structure: contains datablock(s) I, global. DOI: 10.1107/S2053229621003430/vp3014sup1.cif

Structure factors: contains datablock(s) I. DOI: 10.1107/S2053229621003430/vp3014Isup2.hkl

1H NMR spectra. DOI: 10.1107/S2053229621003430/vp3014sup3.pdf

CCDC reference: 1938631

## Figures and Tables

**Table 1:** Experimental details

Crystal data	
Chemical formula	C_9_H_13_N_3_O_4_
*M*_r_	227.22
Crystal system, space group	Orthorhombic, *P*2_1_2_1_2_1_
Temperature (K)	100
*a*, *b*, *c* (Å)	6.8378 (4), 11.4334 (7), 12.7595 (8)
*V* (Å^3^)	997.53 (11)
*Z*	4
Radiation type	Cu *K*α
μ (mm^−1^)	1.02
Crystal size (mm)	0.22 × 0.18 × 0.16

Data collection	
Diffractometer	Bruker APEXII Kappa CCD
Absorption correction	Multi-scan (*SADABS*; Bruker, 2014 ▸)
*T*_min_, *T*_max_	0.75, 0.85
No. of measured, independent and observed [*I* > 2σ(*I*)] reflections	11824, 1768, 1719
*R*_int_	0.037
(sin θ/λ)_max_ (Å^−1^)	0.596

Refinement	
*R*[*F* ^2^ > 2σ(*F* ^2^)], *wR*(*F* ^2^), *S*	0.025, 0.061, 1.12
No. of reflections	1768
No. of parameters	161
H-atom treatment	H atoms treated by a mixture of independent and constrained refinement
Δρ_max_, Δρ_min_ (e Å^−3^)	0.12, −0.18
Absolute structure	Flack *x* determined using 684 quotients [(*I* ^+^) − (*I* ^−^)]/[(*I* ^+^) + (*I* ^−^)] (Parsons *et al.*, 2013 ▸)
Absolute structure parameter	0.04 (9)

Computer programs: *SAINT* (Bruker, 2015 ▸), *SHELXS2014* (Sheldrick, 2015*a* ▸), *SHELXL2014* (Sheldrick, 2015*b* ▸), *APEX3* (Bruker, 2016 ▸) and *XP* (Bruker, 1998 ▸).

**Table 2:** Selected geometric parameters (Å, °)

N1—C1′	1.499 (2)	N4—C4	1.337 (3)
			
C6—N1—C1′	122.33 (16)	O5′—C5′—C4′	112.26 (16)
C2—N1—C1′	117.08 (15)	C1′—O4′—C4′	110.96 (15)
			
C4—N3—C2—O2	−178.13 (18)	C1′—C2′—C3′—C4′	−33.03 (18)
N4—C4—C5—C6	175.05 (18)	C3′—C4′—C5′—O5′	55.9 (2)
C2—N1—C1′—O4′	173.39 (16)		

**Table 3:** Hydrogen-bond geometry (Å, °)

*D*—H⋯*A*	*D*—H	H⋯*A*	*D*⋯*A*	*D*—H⋯*A*
N4—H4*A*⋯O3′^i^	0.86 (3)	2.10 (3)	2.949 (2)	168 (2)
N4—H4*B*⋯O2^ii^	0.93 (3)	2.05 (3)	2.937 (2)	159 (2)
C1′—H1⋯N3^iii^	1.0	2.46	3.317 (3)	144
C3′—H3*A*⋯N3^iv^	1.0	2.53	3.524 (3)	170
O3′—H3⋯O5′^v^	0.94 (3)	1.86 (4)	2.793 (2)	175 (3)
O5′—H5⋯O2^iii^	0.91 (4)	1.88 (3)	2.716 (2)	152 (3)

Symmetry codes: (i) x+{\script{1\over 2}}, -y+{\script{3\over 2}}, -z+1; (ii) -x+2, y+{\script{1\over 2}}, -z+{\script{1\over 2}}; (iii) -x+2, y-{\script{1\over 2}}, -z+{\script{1\over 2}}; (iv) -x+1, y-{\script{1\over 2}}, -z+{\script{1\over 2}}; (v) -x+1, y+{\script{1\over 2}}, -z+{\script{1\over 2}}.

**Table:** 

	Chemical shift/ppm											
	H-1′	H-2′	H-2′′	H-3′	H-4′	H-5′	H-5′′	3′-OH	5′-OH	NH	H5	H6
**1**	6.04 (*dd*)	2.50 (*m*)	1.81 (*dt*)	4.18 (*td*)	4.12 (*td*)	3.38 (*m*)	3.38 (*m*)	5.18 (*d*)	4.82 (*t*)	6.99 (*s*) 7.07 (*s*)	5.69 (*d*)	7.74 (*d*)
**2**	6.15 (*dd*)	2.10 (*ddd*)	1.92 (*ddd*)	4.19 (*ddd*)	3.76 (*td*)	3.54 (*qdd*)	3.54 (*qdd*)	5.18 (*d*)	4.95 (*t*)	7.10 (*s*) 7.15 (*s*)	5.71 (*d*)	7.78 (*d*)

**Table:** 

	Coupling constant/Hz [*J*(H,H)]									Conformation	
	1′2′	1′2′′	2′2′′	2′3′	2′′3′	3′4′	4′5′	4′5′′	5′5′′	%*N*	%*S*
**1**	7.5	2.8	−14.1	5.4	2.3	2.1	4.8	4.8	–	21	79
**2**	7.6	6.0	−13.3	6.0	3.2	3.1	4.0	4.0	−11.8	28	72

Measured in DMSO-*d*_6_ at 298 K; r.m.s. < 0.4 Hz. H-2′= H-2′_β_; H-2′′ = H-2′_α_. For *PSEUROT* (Van Wijk *et al.*, 1999 ▸) calculations, the coupling constants ^3^ *J*(H1′–H2′), ^3^ *J*(H1′–H2′′), ^3^ *J*(H2′–H3′), ^3^ *J*(H2′′–H3′) and ^3^ *J*(H3′–H4′) were used
