# Supplementary material for: The α-d-anomer of 2′-de­oxy­cyti­dine: crystal structure, nucleo­side conformation and Hirshfeld surface analysis
Source: Acta Crystallogr C Struct Chem. 2021 Apr 9;77(Pt 5):202–8. doi: 10.1107/S2053229621003430 (PMC8097964; doi:10.1107/S2053229621003430)
Supplement: Supplementary file 3 [file c-77-00202-sup3.pdf]

Supporting Information

The α-D-Anomer of 2’-deoxycytidine: crystal structure, nucleoside conformation and Hirshfeld surface analysis

Simone Budow-Busse,[a] Yingying Chai,[a] Sebastian Lars Müller,[a] Constantin Daniliuc[b] and Frank Seela*[a,c]

[a]*Laboratory of Bioorganic Chemistry and Chemical Biology, Center for Nanotechnology, Heisenbergstrasse 11, 48149 Münster, Germany,* [b]*Organisch-Chemisches Institut, Westfälische Wilhelms-Universität Münster, Corrensstrasse 40, 48149 Münster, Germany and* [c]*Laboratorium für Organische und Bioorganische Chemie, Institut für Chemie neuer Materialien, Universität Osnabrück, Barbarastrasse 7, 49069 Osnabrück, Germany*

Table of Contents

Figure S1. ^{1}H-NMR spectrum of α-dC (**1**).

Figure S2. ^{1}H-NMR spectrum of β-dC (**2**).

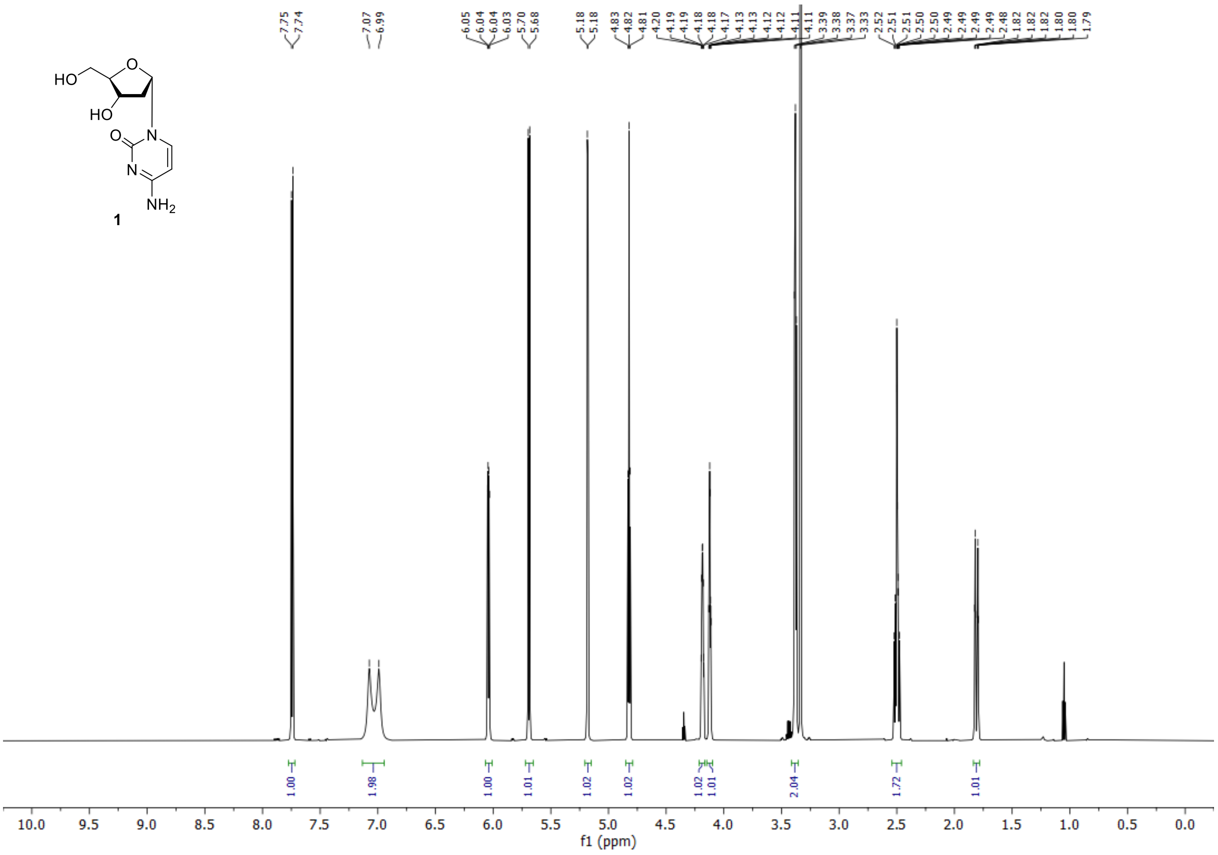

Figure S1. ^{1}H-NMR spectrum of α-2’-deoxycytidine (**1**).

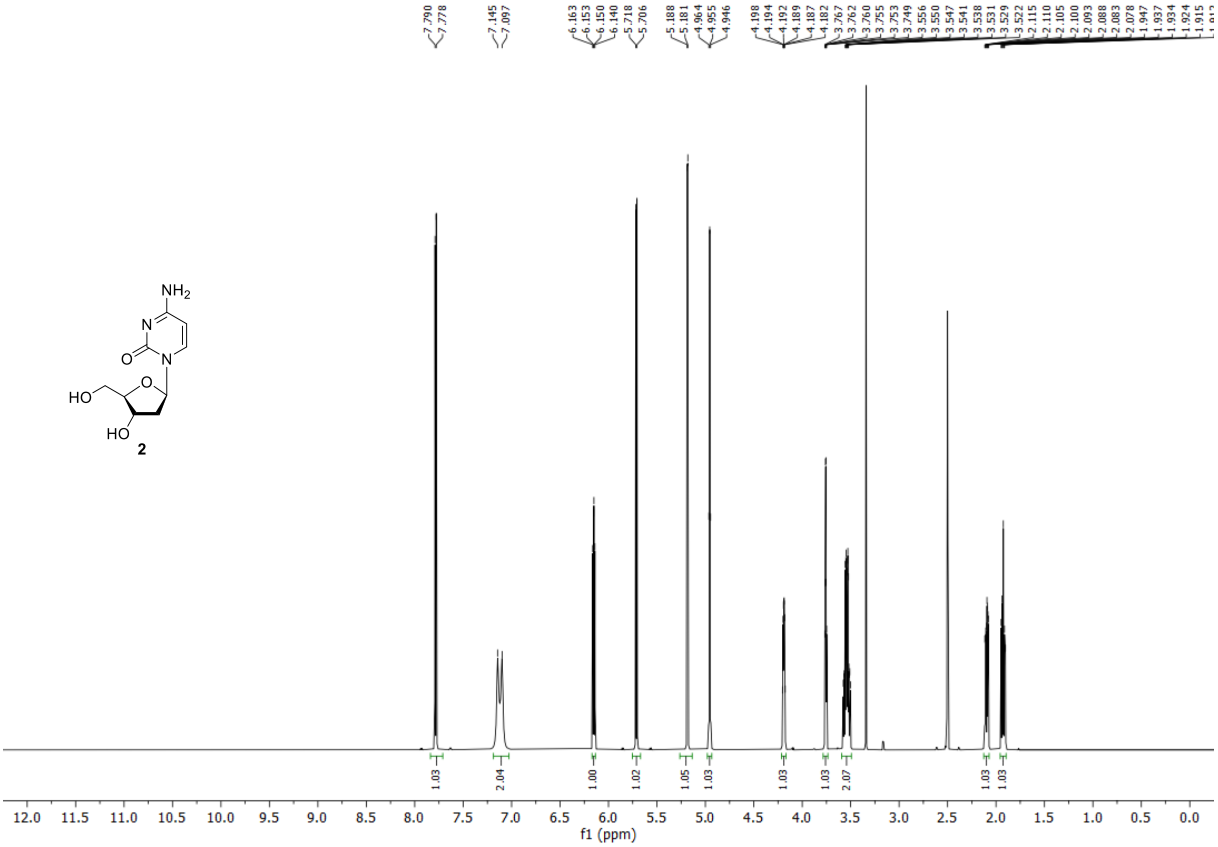

Figure S2. ^{1}H-NMR spectrum of β-2'-deoxycytidine (**2**).
